# Growth, drought response, and climate‐associated genomic structure in whitebark pine in the Sierra Nevada of California

**DOI:** 10.1002/ece3.10072

**Published:** 2023-05-17

**Authors:** Phillip J. van Mantgem, Elizabeth R. Milano, Joan Dudney, Jonathan C. B. Nesmith, Amy G. Vandergast, Harold S. J. Zald

**Affiliations:** ^1^ U.S. Geological Survey Western Ecological Research Center Arcata California USA; ^2^ U.S. Geological Survey Western Ecological Research Center San Diego California USA; ^3^ Environmental Studies Program UC Santa Barbara Santa Barbara California USA; ^4^ Department of Environmental Science, Policy, & Management UC Berkeley Berkeley California USA; ^5^ Department of Plant Sciences University of California Davis California USA; ^6^ USDA Forest Service Pacific Northwest Research Station Portland Oregon USA; ^7^ USDA Forest Service Pacific Northwest Research Station Corvallis Oregon USA; ^8^ Present address: USDA Forest Service Rocky Mountain Research Station Moscow Idaho USA

**Keywords:** climate change, dendrochronology, *Pinus albicaulis*, subalpine forest

## Abstract

Whitebark pine (*Pinus albicaulis* Engelm.) has experienced rapid population declines and is listed as threatened under the Endangered Species Act in the United States. Whitebark pine in the Sierra Nevada of California represents the southernmost end of the species' distribution and, like other portions of its range, faces threats from an introduced pathogen, native bark beetles, and a rapidly warming climate. Beyond these chronic stressors, there is also concern about how this species will respond to acute stressors, such as drought. We present patterns of stem growth from 766 large (average diameter at breast height >25 cm), disease‐free whitebark pine across the Sierra Nevada before and during a recent period of drought. We contextualize growth patterns using population genomic diversity and structure from a subset of 327 trees. Sampled whitebark pine generally had positive to neutral stem growth trends from 1970 to 2011, which was positively correlated with minimum temperature and precipitation. Indices of stem growth during drought years (2012 to 2015) relative to a predrought interval were mostly positive to neutral at our sampled sites. Individual tree growth response phenotypes appeared to be linked to genotypic variation in climate‐associated loci, suggesting that some genotypes can take better advantage of local climatic conditions than others. We speculate that reduced snowpack during the 2012 to 2015 drought years may have lengthened the growing season while retaining sufficient moisture to maintain growth at most study sites. Growth responses may differ under future warming, however, particularly if drought severity increases and modifies interactions with pests and pathogens.

## Graphical abstract

Using extensive surveys across the Sierra Nevada, we show whitebark pine had positive to neutral growth prior to drought in our sampled sites. Growth responses measured in terms of basal area increment remained positive to neutral during drought. Genomic analyses found that individual tree growth response phenotypes appear to be linked to genotypic variation in climate‐associated loci, suggesting that growth is not only controlled by site‐level differences, but that some genotypes can take better advantage of local climatic conditions than others.

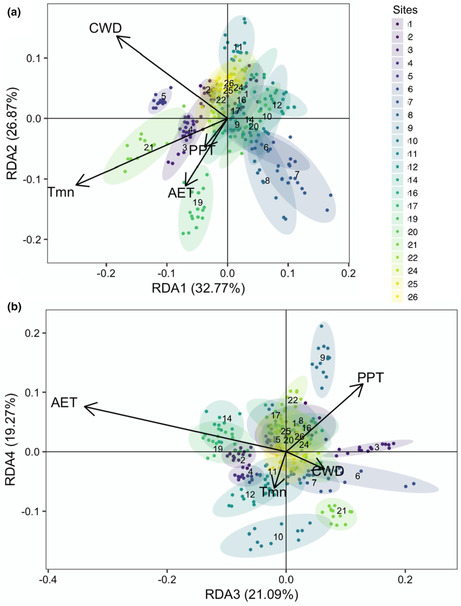

## 1 INTRODUCTION

Whitebark pine (*Pinus albicaulis* Engelm., Pinaceae subgenus Strobus) is a long‐lived tree found in high‐elevation forests of western North America. Because of the multiple roles that whitebark pine plays in subalpine ecosystems, it has been described as a keystone and foundation species. Over much of its range whitebark pine is declining due to a suite of stressors including a non‐native, invasive pathogen (*Cronartium ribicola* J.C. Fisch., the causal agent of white pine blister rust), outbreaks of native pests (mountain pine beetle, *Dendroctonus ponderosae* Hopkins), altered fire regimes, and a rapidly changing climate (Keane et al., 2012; Schwandt et al., 2010; Tomback & Achuff, 2010). As a result, whitebark pine has been listed as an endangered species under the Species at Risk Act (2012) in Canada and is now listed as threatened under the Endangered Species Act in the United States (https://www.regulations.gov/document/FWS‐R6‐ES‐2019‐0054‐0001, accessed 1/6/2023).

The Sierra Nevada of California is the southernmost range extent of whitebark pine, where it experiences warmer, drier conditions relative to other portions of its distribution (Arno & Hoff, 1989). Whitebark pine in the Sierra Nevada is genetically differentiated from other regions, which may reflect adaptations to climatic differences and reduced gene flow (Richardson et al., 2002; Syring et al., 2016). Additionally, whitebark pine in the Sierra Nevada ecoregion of California and Nevada (https://www.epa.gov/eco‐research/ecoregions‐north‐america, accessed 3/9/2022) currently appears to be less impacted by biotic and abiotic stressors relative to other portions of its range. A recent synthesis of whitebark pine status from a US national forest inventory database showed slightly over half of the standing whitebark pine ≥12.5 cm DBH (stem diameter at breast height [1.37 m]) were dead; however, within the Sierra Nevada alone only <10% of stems were dead (Goeking & Izlar, 2018). The lower mortality in the Sierra Nevada likely reflects the lower incidence of stressors. While white pine blister rust has been present in the Sierra Nevada at least since the 1960s (Kinloch, 2003), the proportion of infected trees across the Sierra Nevada is highly variable. Maloney et al. (2012) found that approximately 35% of whitebark pine were infected within the relatively mesic Tahoe Basin, which contrasts with ≤1% of infected whitebark pine in the cooler, drier sites of the central and southern Sierra Nevada (Dudney et al., 2020; Nesmith et al., 2019). There are currently few published reports of mountain pine beetle outbreaks in whitebark pine in the Sierra Nevada (Meyer et al., 2016; Millar et al., 2012). Historical fire return intervals in subalpine forests of the Sierra Nevada are estimated to be greater than a century (Safford & Van de Water, 2014), so fire exclusion over the past century is unlikely to have greatly affected whitebark pine in this ecoregion.

The persistence of whitebark pine may be impacted by ongoing changes in climate (Chang et al., 2014; Keane et al., 2017; McKenney et al., 2007). Subalpine forests are generally thought to be energy limited; as a result, warming has been associated with increasing tree growth, stand densification, and treeline advance (Dolanc, Thorne, et al. 2013; Harsch et al., 2009; Salzer et al., 2009; Trant et al., 2020). In subalpine forests, precipitation often arrives as snow, which can be negatively related to tree growth that year (e.g., Dolanc, Westfall, et al. 2013; but see Graumlich, 1991). However, warming has also led to greater proportions of precipitation arriving as rain, reducing depth, and duration of annual snowpack (Fyfe et al., 2017; Marlier et al., 2017; Mote et al., 2016; Pederson et al., 2011). This could potentially benefit high‐elevation forests in the near‐term, but may lead to severe moisture deficits as warming intensifies under expected future climates (Ullrich et al., 2018). Continued warming may also portend a future with increasing incidence of fire, pest and pathogen activity, and intensified competition (Dolanc, Thorne, et al. 2013; Dudney et al., 2021; Gergel et al., 2017; Harpold, 2016; Millar et al., 2004; Schwartz et al., 2015).

Beyond projected changes in average environmental conditions, extreme events, including droughts, will also likely affect whitebark pine. From 2012 to 2016 California experienced a severe drought, and 2014 to 2015 represented some of the warmest, driest years in the instrumental record (Diffenbaugh et al., 2015; Griffin & Anchukaitis, 2014; Ullrich et al., 2018; Williams et al., 2015). Snow course data in California showed that 2015 had record low April 1 snow water equivalents, particularly at lower elevations (Mote et al., 2016). Range‐wide estimates of snowpack in the Sierra Nevada suggested 2012 to 2015 had low cumulative snowfall, including substantial reductions in snowpack at high elevations in 2015 (Margulis et al., 2016). These conditions caused deep soil drying at low elevations (Goulden & Bales, 2019), leading to vegetation moisture stress and widespread tree mortality (Asner et al., 2016; Young et al., 2017). Tree mortality during and immediately following the drought was concentrated in lower elevation forests and was often driven by interactions with bark beetle activity (Fettig et al., 2019; Stephenson et al., 2019). Pest and pathogen activity is often less severe at high elevations in the Sierra Nevada (Das et al., 2016; Dudney et al., 2020), but it is unclear whether bark beetle activity is changing in these subalpine forests.

Tree growth provides an indication of how individuals respond to drought and changing environmental conditions. Secondary growth, measured by stem diameter, is responsive to environmental conditions (Speer, 2010). Moisture deficit may induce trees to reallocate resources away from noncritical functions, such as growth (Waring, 1987). As a result, short‐term reductions in stem growth may be a symptom of stress for individual trees and can be predictive of mortality (Cailleret et al., 2019; Dudney et al., 2021). Given the broad latitudinal range of whitebark pine in the Sierra Nevada, trees are unlikely to respond uniformly to changing environmental conditions. We expect tree growth to respond to broad latitudinal gradients in climate, while varying with microclimatic differences in elevation, aspect, and soils. Moreover, local or population‐level genetic variation may impact whitebark pine adaptation to climatic conditions (Depardieu et al., 2020; Trujillo‐Moya et al., 2018). Patterns of genetic variation within a species are shaped by both historical and contemporary vicariant and adaptive processes, including selection, seed and pollen dispersal patterns, range expansions and contractions and range fragmentation due to geographic features (Slatkin, 1987; Wright, 1978). Previous range‐wide studies of genetic variation in whitebark pine have shown that the Sierra Nevada is genetically distinct from the rest of the species range (Richardson et al., 2002; Syring et al., 2016). Therefore, gene exchange with the rest of the species distribution is likely limited. While genetic structure of whitebark pine across the entire Sierra Nevada has not been well‐characterized, studies of limited areas within this range reported relatively high levels of genetic similarity among stands (Jorgensen & Hamrick, 1997; Lind et al., 2017; Richardson et al., 2002; Rogers et al., 1999).

In this study, we examined longer‐term patterns in whitebark pine stem growth and interannual climate and estimated stem growth during the recent meteorological drought in the Sierra Nevada. Further, we contextualize growth responses in relation to population genomic structure (genome‐wide SNP analysis). We expected stem growth responses would provide insight into the important climate drivers over longer time periods, while short‐term patterns in radial growth should provide insight into the short‐term growth responses during meteorological drought. Finally, genetic data describe the degree of local population adaptation to a particular site and may provide a genetic perspective of drought vulnerability.

We used growth and genetic data to answer the following questions: (1) how did recent whitebark pine growth respond to variability in interannual climate in the Sierra Nevada? (2) What was the growth response of whitebark pine during the recent meteorological drought years and how did it vary across the Sierra Nevada? and (3) Were growth responses during the drought years linked to climate‐associated genetic variation? By combining measurements of growth and genomic structure over a common set of individual trees and sites, we aim to provide a framework to characterize the environmental and intrinsic genetic drivers of variation in growth. This study potentially provides insight into responses and adaptation of whitebark pine to possible future climatic conditions in its southernmost ecoregion.

## 2 METHODS

### 2.1 Whitebark pine in the Sierra Nevada

**FIGURE 1:**
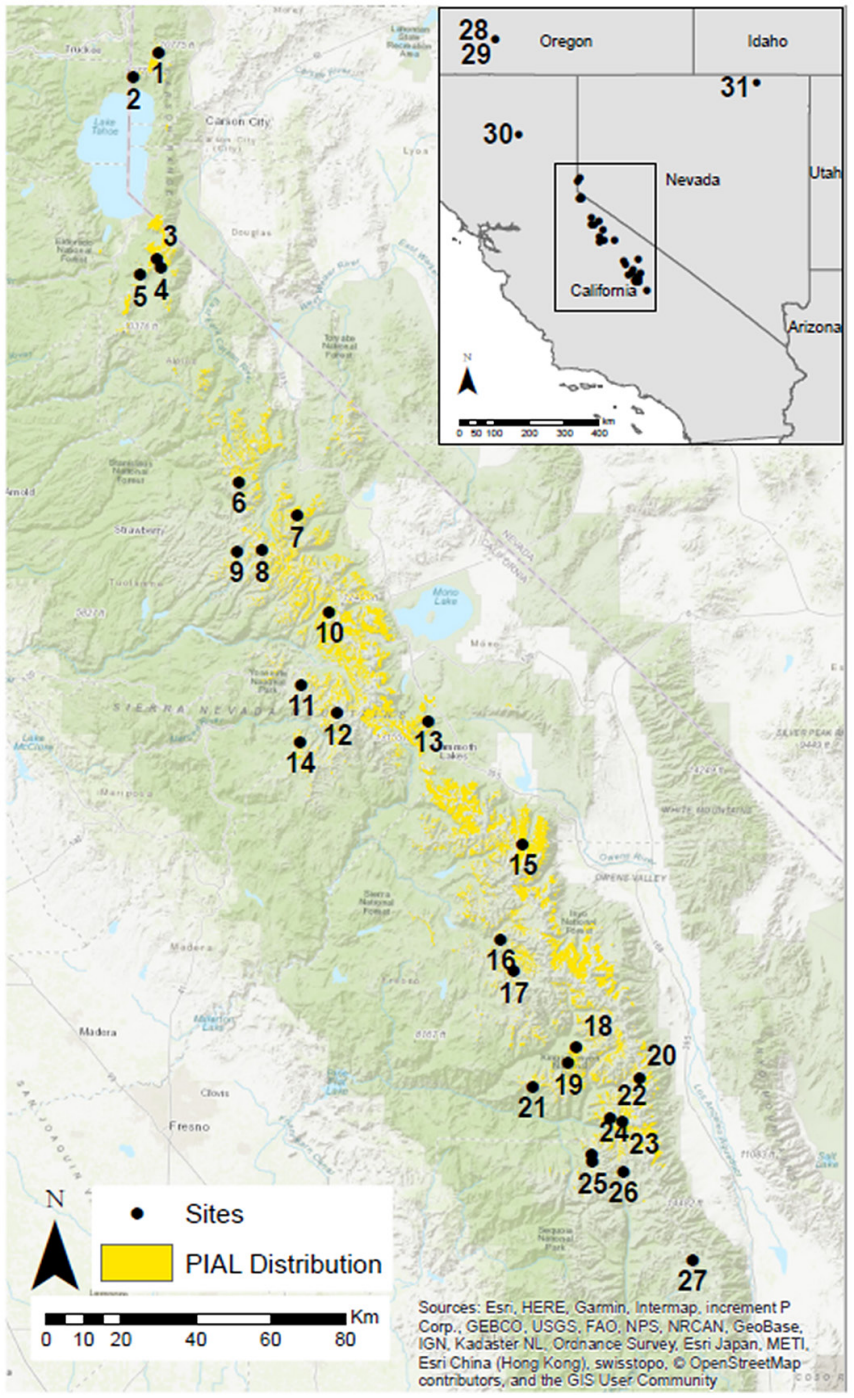
Locations of 27 study sites across the whitebark range in the Sierra Nevada. Yellow area shows the current estimated distribution of whitebark pine (PIAL) in our study area (https://www.fs.usda.gov/detail/r5/landmanagement/gis/?cid=fseprd697598, accessed March 19, 2021). The locations of outgroup populations (sites 28–31) used in genetic analysis are shown in the inset map, with Sierra Nevada sites inside the box. Two outgroup populations at Crater Lake National Park were in close proximity and appear as a single symbol.

Whitebark pine in the Sierra Nevada of California is found from north of the Lake Tahoe basin to south of Sequoia National Park (approximate latitudinal range = 36°–39°; Figure 1). The Sierra Nevada is characterized by a Mediterranean climate with warm, dry summers and cold, wet winters. Whitebark pine grows in the subalpine zone of the Sierra Nevada, where temperatures are low, growing seasons are short (2 to 3 months), and most annual precipitation falls as snow. In the Sierra Nevada, whitebark pine occurs between 2100 and 3700 m (https://www.fs.fed.us/rm/highelevationwhitepines/About/dist.htm#california, accessed 3/10/2022), growing as vertical stems at low elevations and as a more shrub‐like growth form (*krummholz*) near treeline (Millar et al., 2004, 2020). In the Sierra Nevada, whitebark pine often grows in discrete patches where it is the canopy dominant, although it can also grow in mixed stands with other subalpine species (Fites‐Kaufman et al., 2007), including limber pine (*Pinus flexilis* James), lodgepole pine (*P. contorta* Dougl.), foxtail pine (*P. balfouriana* Grev. & Balf.), western white pine (*P. monticola* Douglas ex D. Don), mountain hemlock (*Tsuga mertensiana* (Bong.) Carr.), and red fir (*Abies magnifica* A. Murray bis var. *magnifica*).

Whitebark pine has a mutualistic relationship with a seed‐dispersing bird, Clark's nutcracker (*Nucifraga columbiana*), which promotes a clumped pattern of individual trees and influences population genetic structure from tree to regional scales. Clark's nutcracker creates caches of 1 to 15 seeds (Tomback, 1978, 1982), which may develop into multiple, often genetically distinct, basally fused stems (Linhart & Tomback, 1985; Rogers et al., 1999). Whitebark pine seeds do not have wings and are dependent on the Clark's nutcracker for long‐distance dispersal, with dispersal distances of up to 32.6 km recorded from radiotagged birds along the eastern Cascade mountains (Lorenz et al., 2011). The pollen, however, is wind dispersed (Fryer, 2002) potentially allowing a high degree of genetic mixing across longer distances (Richardson et al., 2002). Even in cases where overall genomic similarity and gene flow are high, variation in allele frequency in certain genes has been noted at local scales. For example, Lind et al. (2017) reported significant differences at loci associated with soil water availability across trees sampled in the Tahoe Basin, suggesting that local adaptation occurs in this species at relatively fine spatial scales despite overall high genomic similarity and outbreeding opportunities provided by the seed caching behavior of Clark's nutcracker and pollen dispersal. Across the species range, Syring et al. (2016) reported a subset of genes with very high differentiation across geographic regions and strong associations with latitude, suggesting that these genes were tracking climatic differences.

### 2.2 Study sites and tree selection

In the summers of 2018 and 2019, we collected tree cores from 27 sites across the range of whitebark pine in the Sierra Nevada (Figure 1). We considered areas that were on average <2 km from randomly located U.S. Forest Service and National Park Service whitebark pine monitoring plots to ensure accessibility and whitebark pine presence in the area (Nesmith et al., 2019; Slaton et al., 2019). Within these areas, we identified three to five polygons that were primarily south‐facing aspects (135°–225°) with slopes <25° using a digital elevation model (Gesch et al., 2002). We stratified these polygons based on contrasting snow accumulation and temperatures from 2012 to 2015 (at least ±1 SD from the mean value across the range of whitebark pine in the Sierra Nevada) estimated from the Basin Characterization Model (BCM; Flint et al., 2021; see Methods, *Climate*). We determined final sampling sites based on crew safety and the presence of multiple non‐*krummholz* whitebark pines. We avoided *krummholz* due to their complex and potentially differing growth responses to environmental conditions (Millar et al., 2020).

Starting from a random point within each site, we selected trees along a transect perpendicular to the slope to maintain consistent aspect and elevation along the transect. In sites where non‐*krummholz* whitebark pine was sparse or where many trees failed to meet selection criteria, most or all individuals within a site were sampled, so trees cannot be considered to be randomly selected. Transect length varied by whitebark pine density, with the transect length extending >1 km where whitebark pine was sparse. Whitebark pine can be extremely difficult to differentiate in the field from other co‐occurring five‐needled pines, such as western white pine and limber pine. We minimized the potential for species misidentification by sampling within areas of known whitebark pine presence, focused on large trees that have field identification characteristics such as male and female cones, and used extremely experienced field crews. To further confirm species identification, we tested a subset of samples using a molecular assay that distinguishes whitebark from limber pine based on differences in chloroplast genes (see Alongi et al., 2019 for methods). All samples were confirmed as whitebark pine.

### 2.3 Field collections

We collected 800 tree cores. After removing duplicate and unmeasurable cores (see below) along with one core from a tree with symptoms of white pine blister rust, we had 766 cores used in analysis. At each site, we had an average of 28 individual whitebark pines with usable tree core data (range = 17 to 30 trees). Sampled trees were required to be growing upright, free of significant damage, disease, and beetle activity. Stem diameters for sampled trees ranged from 6.3 to 89.0 cm DBH (average = 25.2 cm), and tree heights ranged from 2.3 to 22.0 m (average height = 7.3 m). There were five trees with missing DBH measurements and two trees with missing height measurements. One sampled tree had symptoms of white pine blister rust and was removed from analysis of growth but was retained for the genetics analyses. If stems were clumped, we cored only one arbitrarily selected stem per clump. Needle samples were collected from cored trees for genetic analysis (NPS Permit #: YOSE‐2019‐SCI‐0061). We were working largely in wilderness areas, and the removal of cone‐bearing branches for herbarium specimens would have been impractical.

### 2.4 Climate data

**FIGURE 2:**
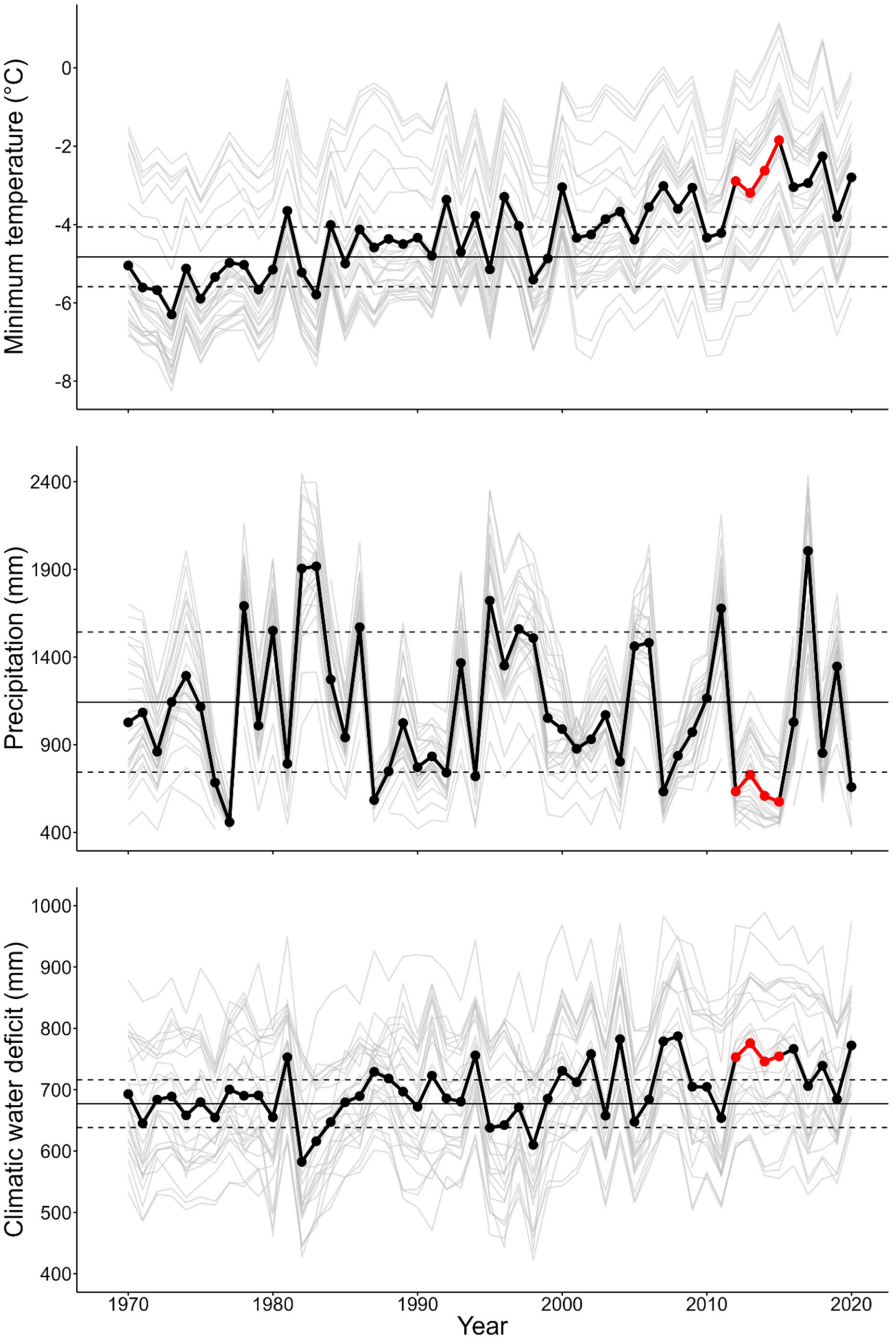
Average annual minimum temperature, precipitation, and climatic water deficit for WY 1970 to WY 2020 from the Basin Characterization Model across the 27 sampling sites in the Sierra Nevada. The black line shows the among‐site annual average, with the gray lines showing individual site values. The horizontal lines represent the average annual values from WY1970 to WY1999, with dashed lines showing ±1 SD. The years WY2012 to WY2015 (drought years) are shown in red, where annual precipitation was <1 SD below the WY1970 to WY1999 average.

For the centroid of each sampling site, we obtained BCM climate estimates for water years (WY, October to September) 1970 to 2020 (https://www.sciencebase.gov/catalog/item/5f29c62d82cef313ed9edb39). Data for 26 sites were accessed on 11/12/2020, with data for one site accessed on 9/22/2021. BCM uses gridded instrumental climate products from the Parameter Elevation Regression on Independent Slopes Model (PRISM, Daly et al., 2008) to run a downscaled (270 m) water balance model, estimating monthly minimum temperature (Tmn), maximum temperature (Tmx), precipitation (PPT), potential evapotranspiration (PET), actual evapotranspiration (AET), and climatic water deficit (PET–AET = CWD). We used the BCM data to calculate 30‐year normal climate values for each site from WY1970 to WY1999. Across our study sites, BCM outputs indicate that from WY1970 to WY2020 annual minimum temperature was increasing, with variable precipitation and gradually increasing climatic water deficit (Figure 2). Plots of water year precipitation totals suggested WY2012 to WY2015 were particularly dry at our sites (<1 SD from mean annual precipitation from WY1970 to WY1999). We found the years 2012 to 2015 to have low April 1 snow water equivalents (SWE) at SNOwpack TELemetry (SNOTEL) stations close to our sites (https://wcc.sc.egov.usda.gov/nwcc/rgrpt?report=swe_hist, accessed 10/30/ 2021) (note that SNOTEL data are captured in the PRISM estimates that are used by the BCM). We considered the period from WY2012 to WY2015 to be “drought years” at our sites, although statewide the drought is described as lasting through 2016 (Ullrich et al., 2018).

### 2.5 Sample processing

#### 2.5.1 Tree cores

Following standard sample preparation procedures (Speer, 2010), tree cores were glued to wooden mounting sticks and sanded with progressively finer sandpaper (up to 800 grit) until ring boundaries were clearly delineated. Ring widths were measured using a calibrated color flatbed scanner (2400 dpi) and WinDENDRO version 2017a software (Regent Instruments Canada Inc.). Cores with ring widths too narrow to be visualized with the flatbed scanner were measured using a 7–45× stereo microscope, Unislide TA tree‐ring measuring system (Velmex Inc.), and Measure J2X software (VoorTech Consulting). All ring widths were measured to the nearest 0.001 mm. Series were cross‐dated to ensure correct calendar year assignment of each growth ring using a multistage process, because whitebark pine samples had numerous small or missing rings. First, series were visually compared within each sampling location to identify potential marker years (i.e., years of notably low or high growth). Second, series within each sampling location were statistically cross‐dated following standard dendrochronology procedures using the dplR package for R (Bunn, 2008). Third, internally cross‐dated series were statistically cross‐dated against a recently developed regional chronology for whitebark pine (Millar et al., 2012).

#### 2.5.2 Population genomic data collection

Needle tissue for DNA analysis was collected from each sampled tree. Additional samples were provided from opportunistically collected needles at Crater Lake National Park, Lassen Volcanic National Park (Klamath Inventory and Monitoring Network, National Park Service), and the Jarbidge Mountains in northeastern Nevada (University of Nevada, Reno), to act as outgroup sites for genetic analysis. For each tree, five‐needle fascicles were placed into coin envelopes with two 5‐gram silica packets. Upon return from the field site, silica packets were removed, and envelopes were transferred to a 45°C oven to accelerate desiccation and aid in DNA preservation (Bainard et al., 2010; Chase & Hills, 1991; Doyle & Dickson, 1987) and stored in silica gel desiccant prior to extraction.

Genomic DNA was extracted from dried needles using the E‐Z 96® Plant DNA Kit (Omega Bio‐tek Inc., Norcross, Georgia) with Antifoam Y‐30 (Sigma‐Aldrich, St. Louis, Missouri) added to the homogenizer plate and a 60‐min room temperature incubation before elution in 100 μL of water. Reduced representation genomic libraries were prepared using a modified double‐digest restriction‐site associated DNA sequencing (ddRAD) scheme (Peterson et al., 2012) in which 700 ng of DNA was digested with EcoRI and MseI restriction enzymes (New England Biolabs, Ipswich, Massachusetts). In‐line barcodes were ligated onto the EcoRI cut site, and fragments were size‐selected on a Pippin Prep (Sage Science, Inc., Beverly, Massachusetts) at a 400 ± 30 bp range. Each library of eight multiplexed samples was PCR‐amplified for 15 cycles with the addition of a unique library index sequence. Libraries were quantified, pooled, and sequenced at the University of Oregon Genomics and Cell Characterization Core Facility (Eugene, Oregon) using an Illumina HiSeq 4000 to generate 100 bp single‐end reads (Illumina, Inc., San Diego, California). Forty‐eight samples were processed per lane. Raw sequence reads were demultiplexed, trimmed, and filtered for quality using the process_radtags program in Stacks v2.53 (Catchen et al., 2013). Cleaned reads were aligned to the *Pinus lambertiana* v1.5 genome (Crepeau et al., 2017) using *BWA mem* (Li & Durbin, 2010) and genotyped using the ref_map.pl program in Stacks v2.53 (Catchen et al., 2013). Final filtering steps included removing samples with an average read depth < 9×, removing loci not present in at least 80% of samples, and selecting a single SNP per locus. All computation was performed on the USGS Yeti high‐performance computing cluster. Data for our study are available on ScienceBase (van Mantgem et al., 2022), and raw sequence reads are available as NCBI BioProject ID PRJNA917690 (https://www.ncbi.nlm.nih.gov/bioproject/917690).

### 2.6 Analyses

#### 2.6.1 Recent stem growth

To describe patterns in recent stem growth prior to the drought years, we focused on 1970 to 2011. This interval covers both wet and dry years in California, including the 1977 drought (Griffin & Anchukaitis, 2014). Summary statistics of ring‐width series were calculated for each study site. Summary statistics include the number of series, number of tree rings, the first and last year of series, average series length, average autoregressive coefficient (AR1, first‐order autocorrelation, estimating correlation between growth in a given year versus the preceding year), and average interseries correlation (i.e., degree that tree growth is synchronous). To examine growth trends, tree‐ring series were converted to annual basal area increment (BAI) based on the diameter of each tree and the width of each ring moving toward the pith of the tree. BAI series were normalized by their standard deviations (sBAI) to adjust for differences in BAI between large and small trees. Following normalization, series sBAI values were filtered to the 1970 to 2011 period. Trends of sBAI for the 1970 to 2011 period were calculated for each tree as the linear regression slope coefficient of sBAI over time. Overall site‐level trends were determined by assessing the 95% confidence interval (CI) of individual tree sBAI slope coefficients within sites. Where the lower 95% CI of the slope coefficients were >0, the site was considered to have positive growth trends, while sites where the upper 95% CI of the slope coefficient was <0 were considered to have negative growth trends. We considered sites where 95% CIs overlapped 0 as having neutral growth trends. To gauge the overall pattern in growth trends, we tallied positive and negative average site sBAI trends where 95% CIs did not overlap 0.

We related site‐level mean annual sBAI from 1971 to 2011 to site‐level water year annual climate and 1‐year lagged climate variables using linear regression. We excluded observations from 1970 to include lagged climate variables. Models included an AR1 correlation structure to account for correlation in model error terms in adjacent years. We standardized all model predictors by subtracting the mean and dividing by the standard deviation, allowing model effects to be more easily compared. We selected the model with the most support using the Akaike Information Criterion, corrected for small sample size (AICc, Burnham & Anderson, 2004). Models with ΔAICc values of <4 were considered to have similar support. Model fits were evaluated with a pseudo *r* ^2^ calculated by squaring the correlation of the fitted versus observed growth. Models were constructed using the *nlme* package in R (Pinheiro et al., 2020; R Core Team, 2021).

#### 2.6.2 Stem growth during drought years

We created a growth response index to the meteorological drought analogous to the resistance index of Lloret et al. (2011). The stem growth drought response index for individual trees was calculated by dividing average BAI during the meteorological drought years (2012 to 2015) by average BAI over the same number of years prior to the meteorological drought years (2008 to 2011). For the BAI response index, values >1 represent greater growth during the drought years relative to the predrought period, while values <1 represent lower stem growth.

We checked whether the BAI drought response index was sensitive to the years we used to define the drought and predrought periods (Schwarz et al., 2020), finding similar responses using longer drought (2012 to 2016) and predrought (2007 to 2011) intervals. We calculated site‐level averages and 95% CIs for the BAI drought response index. As with BAI trends, overall response for each index was determined by tallying positive and negative average response indices where the site‐level 95% CIs did not overlap 0. We considered sites where 95% CIs overlapped 0 as having a neutral response. We correlated site‐level average response indices with averaged standardized climatic anomalies during the drought years, calculated as average of the annual site‐level *z*‐scores from WY2012 to WY2015 (drought years) relative to WY1970 to WY2015. We created average *z*‐scores for Tmn, PPT, and CWD for correlations with the BAI drought response index. Where the data had a large departure from a normal distribution, we used the Spearman rank correlation (*r*_*s*_).

#### 2.6.3 Genomic population structure and climate associations

Genetic differentiation among sampling sites (*F*_ST_) was calculated using the populations program in Stacks, while filtering for one biallelic single‐nucleotide polymorphism (SNP) per locus (detailed in Hohenlohe et al., 2010). We calculated unbiased expected heterozygosity (uHe) on the filtered SNP dataset in dartR v. 2.0.4 (Mijangos et al., 2022). Nucleotide diversity (*π*) was calculated on all variable sites within loci using the haplotype‐wise filter, kernel smoothing, and bootstrap resampling flags in the populations program in Stacks. Two genetic clustering analyses were used to explore the pattern of genetic structure among individuals. First, a principal component analysis (PCA) was implemented in adegenet v2.1.1 (Jombart, 2008) to visualize the genotypes in multidimensional space. Second, FastSTRUCTURE v1.0, a Bayesian clustering method (Raj et al., 2014), was run for values of K (number of genetic clusters) from 1 to 9 to identify differences in allele frequencies across populations. A range of optimal *K* values was determined using the chooseK.py script in the fastSTRUCTURE package. We tested for a pattern of increasing genetic differentiation (*F*_ST_) with increasing geographic distance among Sierra Nevada study sites using a Mantel test for matrix correlation (Mantel, 1967) with significance assessed by 999 permutations of the genetic differentiation matrix. Samples from outgroup sites were included in clustering analyses but excluded from environmental association analysis, since this study is focused on associations across Sierra Nevada study sites.

We used a redundancy analysis (RDA; van den Wollenberg, 1977) to determine how groups of loci covary in response to climate in a multivariate framework. RDA is a two‐step approach that first uses multiple linear regression of response variables (genotypes) on predictor variables (climate variables). Second, a matrix of the fitted values of all response values generated through multiple linear regression is subject to PCA. RDA is a sensitive method for determining combinations of multiple loci that are associated with environmental variables (Forester et al., 2018). Each locus may be of small individual effect, but together they may confer a selective advantage to the associated environmental conditions. Analyses were performed in the *vegan* v2.5‐6 R package, as outlined in Forester et al. (2018), using individual genotype data and site‐level climate data.

We used BCM 30‐year normals (WY1970 to WY1999) for annual Tmn, Tmx, PPT, PET, AET, and CWD in genetic‐environment multilocus association tests. Although CWD is derived from PET and AET, we had no a priori reason to suspect that any one variable would show stronger associations with genetic data. Climate variables were subject to a forward selection process and variance inflation factor (VIF) cutoff of 10 to remove variables that were highly correlated, resulting in four climate variables to include in the RDA model: Tmn, PPT, AET, and CWD. Because of strong latitudinal gradients in overall genetic differentiation (see results) and temperature and rainfall gradients, we performed a partial RDA with the selected climate variables as predictors and a conditional matrix of plot geographic locations, to control for distance‐based effects. Permutation tests (*n* = 999) were used to assess the significance of the full model, each RDA axis, and the marginal effects of each predictor term and *r* ^2^ was adjusted based on the number of predictor variables. Loci were considered outliers if they were greater than three standard deviations away from the mean RDA axis score. We examined genetic diversity in outlier loci across the range and calculated correlations among individual multilocus genotype RDA loadings and the individual tree BAI drought response indices. These individual‐level measurements were not normally distributed (and transformations did not adequately normalize these data), so we used the Spearman rank correlation to determine relationships.

## 3 RESULTS

### 3.1 Recent stem growth

Across all tree‐ring series, there was high variability within series length, mean series length, first‐order autocorrelation, and mean interseries correlation (Table 1). Mean series length across all sampling locations was 129 years, with site‐level means ranging from 45 to 235 years. Mean first‐order autocorrelation (estimates of the correlation between growth in a given year versus the preceding year) among all sites was 0.63 and ranged from 0.49 to 0.76, indicating that growth was strongly associated with prior year's growth. Mean interseries correlation (synchronicity of tree growth) across all sites was 0.20 and ranged from 0.20 to 0.45, indicating interannual variability of growth was not synchronous across sites or within some sites. Individual‐level sBAI growth trends were negatively correlated with stem diameter (*r*_*s*_ = −.36, *p* < .01).

**TABLE 1:** Overall summary statistics of tree‐ring data by sampling site.

Site	*N* series	*N* rings	Mean series length	Start year	Mean AR1 (SD)	Mean interseries correlation (SD)
1	23	5269	229	1545	0.72 (0.22)	0.2 (0.12)
2	30	1727	58	1876	0.55 (0.17)	0.27 (0.26)
3	26	4896	188	1681	0.72 (0.12)	0.2 (0.1)
4	30	5472	182	1618	0.68 (0.15)	0.26 (0.14)
5	30	3800	127	1768	0.66 (0.12)	0.31 (0.15)
6	30	3295	110	1717	0.58 (0.25)	0.34 (0.18)
7	30	5626	188	1614	0.62 (0.26)	0.25 (0.15)
8	30	3923	131	1694	0.66 (0.15)	0.3 (0.14)
9	29	4805	166	1576	0.62 (0.2)	0.25 (0.13)
10	30	2875	96	1803	0.65 (0.18)	0.29 (0.18)
11	30	5542	185	1669	0.65 (0.2)	0.22 (0.15)
12	30	2299	77	1870	0.58 (0.15)	0.34 (0.18)
13	25	1136	45	1948	0.56 (0.2)	0.44 (0.24)
14	24	3137	131	1758	0.49 (0.27)	0.31 (0.19)
15	25	3245	130	1770	0.74 (0.09)	0.31 (0.14)
16	30	2201	73	1891	0.54 (0.19)	0.4 (0.11)
17	29	2665	92	1738	0.59 (0.15)	0.36 (0.12)
18	30	4564	152	1704	0.72 (0.16)	0.34 (0.14)
19	29	4836	167	1580	0.72 (0.14)	0.3 (0.16)
20	30	3075	103	1868	0.65 (0.18)	0.45 (0.17)
21	30	4497	150	1702	0.62 (0.18)	0.22 (0.17)
22	29	2580	89	1847	0.52 (0.21)	0.36 (0.17)
23	30	3506	117	1723	0.57 (0.19)	0.39 (0.18)
24	30	4340	145	1688	0.62 (0.22)	0.29 (0.13)
25	30	2522	84	1696	0.57 (0.22)	0.33 (0.16)
26	30	2821	94	1843	0.58 (0.19)	0.4 (0.16)
27	17	3996	235	1540	0.76 (0.1)	0.34 (0.14)
All	766	98,650	129	1540	0.63 (0.19)	0.2 (0.17)

*Note*: *N* series, number of tree cores; *N* rings, total number of tree rings; Mean series length, mean number of rings per series; Start year, earliest calendar year captured at the site; Mean AR1, mean first‐order autocorrelation coefficient and 1 standard deviation; Mean interseries correlation, mean pairwise Spearman correlation coefficient between series AR1 = first‐order autocorrelation and 1 standard deviation. The final line of the table shows the summed number of series and rings, the overall mean series length, the minimum start year, and overall average AR1, and overall average interseries correlation across all cores.

**FIGURE 3:**
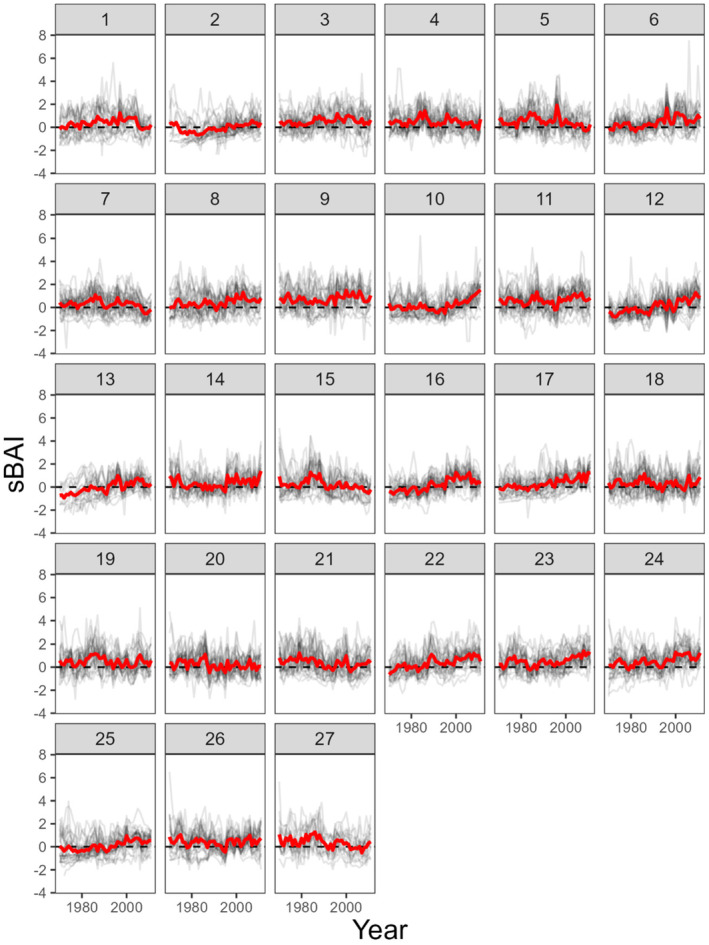
Trends in standardized basal area increment (sBAI) by site for 1970 to 2011. Gray lines are growth trends for individual trees, with the solid red line representing mean sBAI trend at each site.

From 1970 to 2011, sBAI showed high variance among sites (Figure 3), with growth trends visually apparent for some sites (e.g., sites 12, 17, 22, and 23). We identified clear sBAI trends where 95% CIs for parameters of linear models for site‐level growth trend did not overlap 0. Using this criterion, we found 12 sites (44%) had a positive sBAI growth trend, 12 sites (44%) had no clear growth trend (95% CIs overlapped 0), and three sites (11%) sites had a negative growth trend. The model with the most support by AICc for describing environmental influences on site‐level average annual sBAI from 1971 to 2011 included water year Tmn and PPT (Table 2). The strength of the relationship with site‐level average annual sBAI was greater for water year Tmn relative to PPT (scaled estimate ± SE, *β*_Tmn_ = 0.15 ± 0.02, *β*_PPT_ = 0.05 ± 0.01). Site‐level average annual sBAI was not related to CWD (*p* = .42), and models that used CWD had less support relative to models that included independent terms for Tmn and PPT (ΔAICc ≥ 69). While the model with independent terms for Tmn and PPT had the most support by AICc, it explained very little variation in annual site‐level average annual sBAI, with pseudo *r* ^2^ = .03.

**TABLE 2:** Model selection for models of site‐level average annual sBAI.

Model	AICc	ΔAICc	Weight
Ttmn + PPT	644.5	0	0.6
Tmn + PPT + PPT.lag1	645.2	0.7	0.4
Tmn + PPT + Tmn.lag1	651.1	6.6	0
Ttmn + PPT + PPT.lag1 + Tmn.lag1	653.1	8.6	0
Tmn	660.1	15.6	0
Intercept only	705.8	61.3	0
PPT	706.4	61.8	0

*Note*: Models included terms for water year annual minimum temperature (Tmn), precipitation (PPT), 1‐year lagged minimum temperature (Tmn.lag1), and 1‐year lagged precipitation (PPT.lag1). All models included a first‐order autocorrelation term.

### 3.2 Stem growth during drought years

**FIGURE 4:**
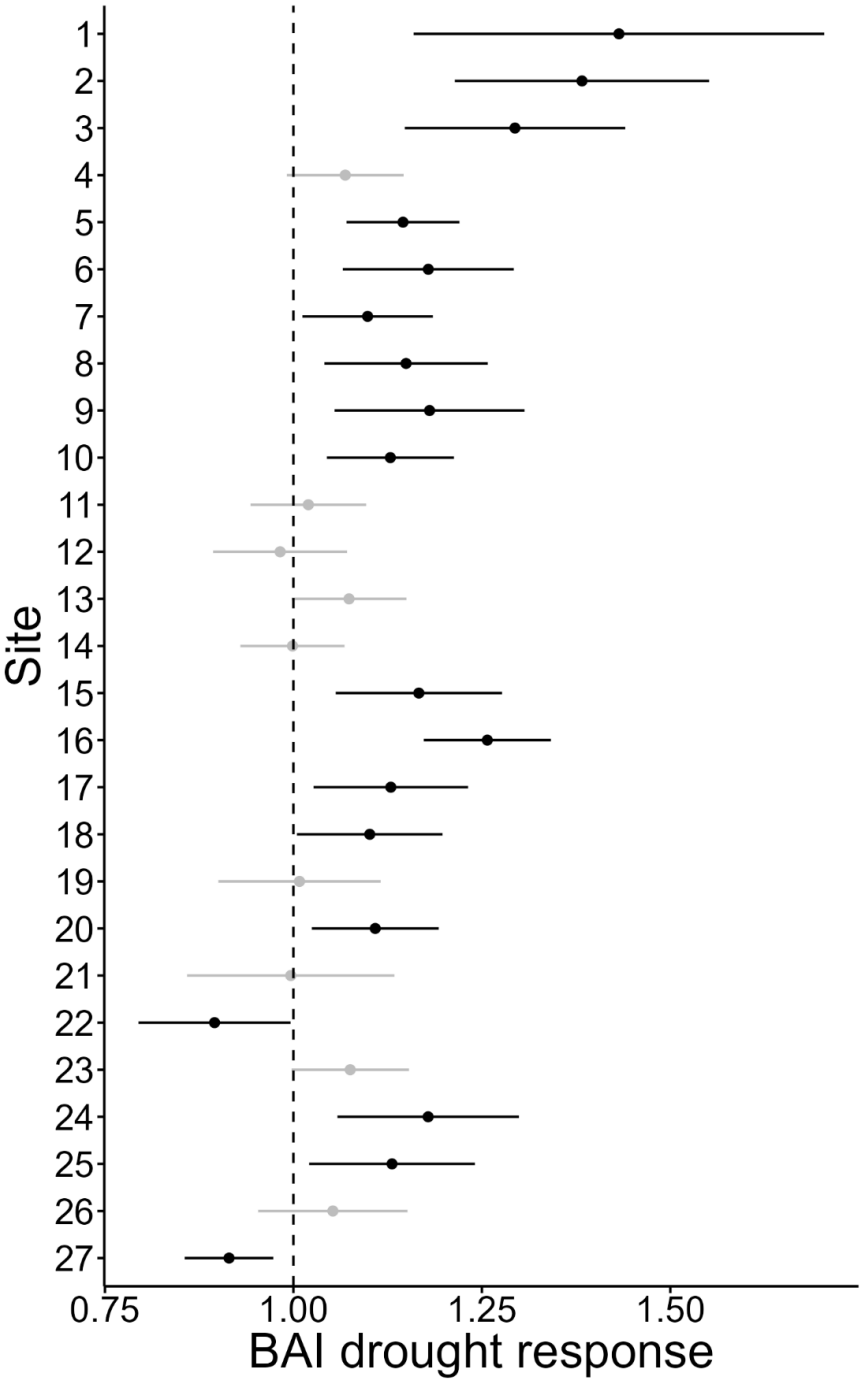
Basal area increment (BAI) drought response by site. Points represent mean site‐level growth response with 95% CIs. The vertical dashed line at 1 represents no change in average growth between drought years (2012 to 2015) relative to the 4 years prior (2008 to 2011). Values greater than 1 indicate greater average growth during the drought years. Black points represent sites where 95% CIs did not overlap 1, gray points represent sites where 95% CIs overlapped 1. Sites are arranged from north (top) to south (bottom).

Site‐level BAI drought response indices were generally positive (Figure 4), with 22 of 27 sites (81%) displaying greater mean BAI during the drought years compared to 4 years preceding drought (growth response metric >1). However, high within‐site variability meant that only 16 sites (59%) displayed clearly positive BAI responses (lower 95% CI >1), while 9 sites (33%) did not display clear BAI responses. Two sites (7%) had a clear indication of negative average BAI growth response (upper 95% CI <1). Site‐level average BAI drought response was not related to site‐level sBAI trend prior to the drought years (*r* = .19, *p* = .33). There appeared to be a latitudinal gradient in average BAI drought response (*r* = .63, *p* < .01), with two northern sites having the greatest BAI drought response. Site‐level average BAI drought response was also correlated with average water year PPT and CWD *z*‐scores during the drought years (PPT, *r* = .60, *p* < .01; CWD, *r* = −.68, *p* < .01). The correlation between BAI drought response and Tmn was not statistically significant.

### 3.3 Genomic data quality and diversity

We estimated population genomic diversity and structure across our sites. Raw sequence reads totaled 3,560,632,042 with a mean of 3,724,485 ± 1,935,474 reads per individual. Mean read coverage per locus was 10.9× ± 1.6× after individuals with an average read count below 9.0× were dropped. Two sites from Sequoia National Park (sites 18 and 23) and three sites from the eastern slopes of the Sierra Nevada (sites 13, 15, and 27) were dropped from further genomic analysis due to low average read depth, so the final genomic dataset included 327 individuals from 22 Sierra Nevada sites and four outgroup sites. The final filtered genetic marker dataset consisted of 44,287 independent loci with 342,375 variant sites. Reducing to a single SNP per locus resulted in 44,287 independent SNPs, 20% overall missing data, and a minimum minor allele frequency of 0.02. More stringent filtering using a minimum minor allele frequency of 0.05 and/or removing individuals with more than 30% missing data slightly reduced the number of SNPs (<1%) and reduced the number of individuals (20%), but had little to no effect on population structure or genetic diversity estimates (Table S1). Therefore, we used the full dataset for all final analyses.

**FIGURE 5:**
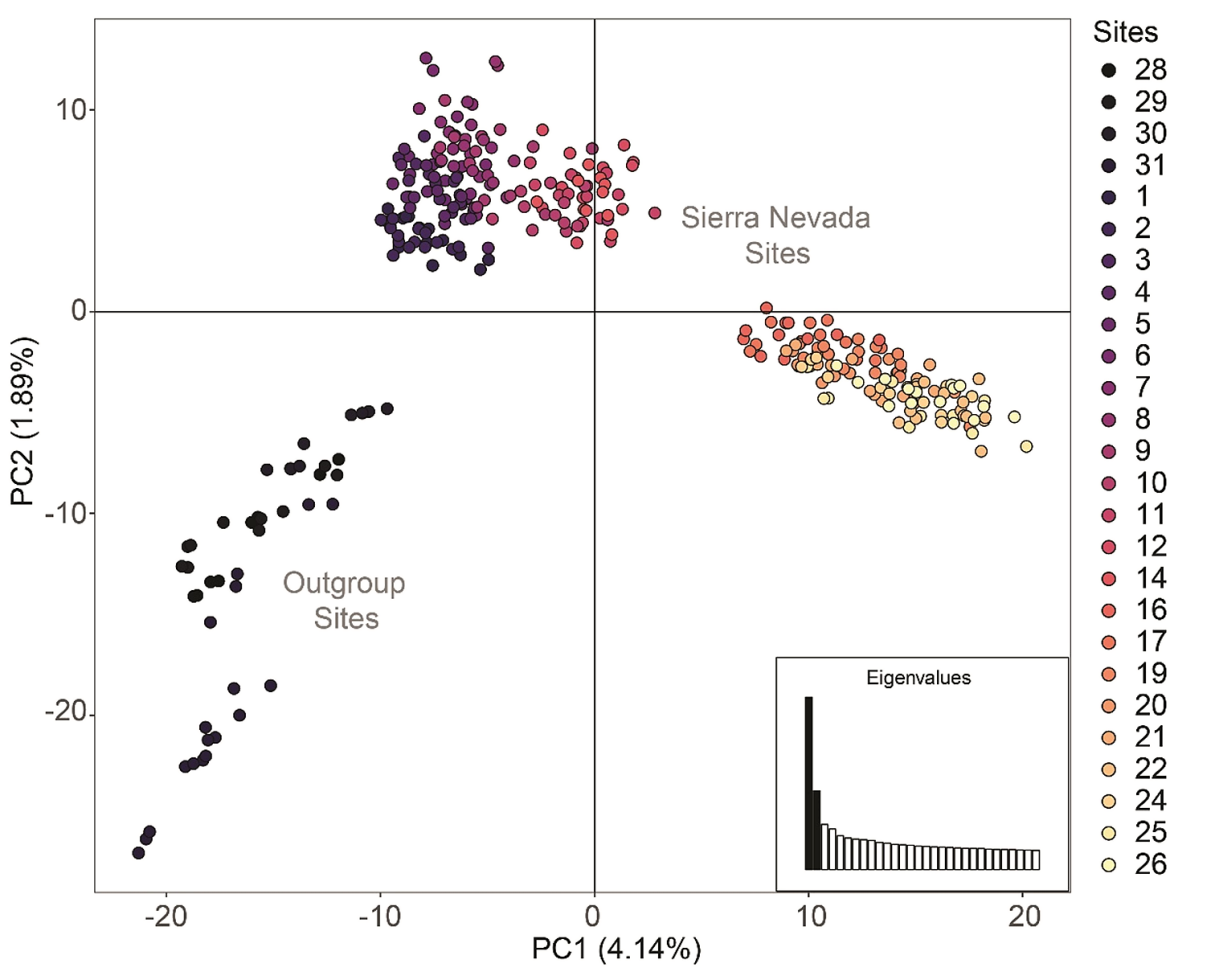
(a) Principal component analysis of whitebark pine sampling locations including Sierra Nevada sites and outgroup sites from Crater Lake National Park, Lassen National Park, and Eastern Nevada. Each point represents an individual, each color indicates site identity, and color gradient corresponds to site latitude (north to south; dark to light). Inset shows relative values of eigenvectors. (b) FastSTRUCTURE barplots where each bar represents an individual and each color represents identity to one of K genetic clusters. Sites are separated by vertical black bars and ordered by latitude (north to south; left to right).

**FIGURE 6:**
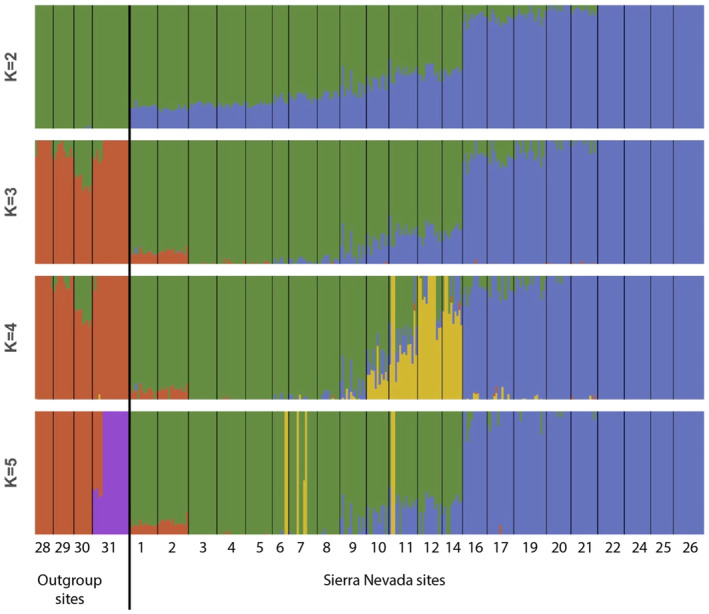
FastSTRUCTURE (Raj et al., 2014) barplots for *K* values between 2 and 5, where each bar represents an individual and each color represents identity to one of *K* genetic clusters. Sites are separated by vertical black bars and ordered by latitude (north to south; left to right).

Using the full dataset, we found minimal population structure across the Sierra Nevada and among outgroup sites. Sierra Nevada sites clustered separately from outgroup sites, further confirming that the Sierra Nevada represents a unique genetic cluster of whitebark pine (Figure 5). There was a predominant latitudinal geographical trend among Sierra Nevada sites along the primary coordinate (4.14% of total variation, Figure 6), with some separation of southern sites in Sequoia National Park from sites further north, corresponding with a geographic break in sampling. FastSTRUCTURE analysis indicated the optimal number of genetic clusters (*K*) ranged from 3 to 5. At *K* = 3 and *K* = 4, we found the outgroup sites form a discrete cluster and the Sierra Nevada sites show a distinct geographical pattern from north to south, and *K* = 5 further separated the Jarbidge Mountains site from the remainder of the outgroup sites (Figure 6). Estimates of genetic differentiation were low among all Sierra Nevada sites (global *F*_ST_ = 0.068, 95% CIs = 0.067 to 0.070; pairwise *F*_ST_ range = 0.004 to 0.088). We detected a significant pattern of genetic isolation by geographic distance (*r* = .7995, *p* < .001) indicating a stepping‐stone structure. Expected heterozygosity and nucleotide diversity were correlated with latitude (highest in Lake Tahoe and lowest in Sequoia NP [uH_e_: *r* = .453, *p* < .034; *π*: *r* = .891, *p* < .001]; Table 3).

**TABLE 3:** Genetic diversity indices for all loci and RDA outlier loci summarized by site.

Site	Sample size	All loci		RDA identified outlier loci	
		uH_e_ (SE)	*π* (SE)	uH_e_ (SE)	*π* (SE)
1	10.77	0.2819 (0.0527)	0.1321 (0.0003)	0.4079 (0.0380)	0.1668 (0.0020)
2	12.06	0.2795 (0.0499)	0.1316 (0.0003)	0.4100 (0.0361)	0.1678 (0.0020)
3	10.98	0.2821 (0.0527)	0.1312 (0.0003)	0.4096 (0.0393)	0.1673 (0.0020)
4	11.18	0.2841 (0.0512)	0.1321 (0.0003)	0.4174 (0.0359)	0.1703 (0.0020)
5	10.47	0.2811 (0.0544)	0.1306 (0.0003)	0.4080 (0.0404)	0.1673 (0.0020)
6	6.45	0.2719 (0.0778)	0.1277 (0.0003)	0.3816 (0.0671)	0.1582 (0.0021)
7	12.03	0.2849 (0.0498)	0.1326 (0.0003)	0.4063 (0.0359)	0.1656 (0.0020)
8	9.03	0.2771 (0.0614)	0.1292 (0.0003)	0.3976 (0.0474)	0.1606 (0.0020)
9	11.11	0.2878 (0.0515)	0.1334 (0.0003)	0.4193 (0.0351)	0.1701 (0.0020)
10	8.16	0.2890 (0.0620)	0.1304 (0.0003)	0.4220 (0.0443)	0.1661 (0.0021)
11	11.23	0.2845 (0.0518)	0.1291 (0.0003)	0.4081 (0.0370)	0.1606 (0.0020)
12	9.86	0.2772 (0.0583)	0.1260 (0.0003)	0.3997 (0.0448)	0.1584 (0.0020)
14	8.27	0.2751 (0.0647)	0.1258 (0.0003)	0.3899 (0.0503)	0.1536 (0.0020)
16	8.20	0.2769 (0.0654)	0.1209 (0.0003)	0.4060 (0.0497)	0.1552 (0.0020)
17	10.35	0.2817 (0.0556)	0.1241 (0.0003)	0.4103 (0.0398)	0.1601 (0.0020)
19	13.81	0.2841 (0.0461)	0.1260 (0.0003)	0.4047 (0.0341)	0.1620 (0.0020)
20	8.94	0.2777 (0.0619)	0.1217 (0.0003)	0.4020 (0.0468)	0.1585 (0.0021)
21	10.75	0.2754 (0.0555)	0.1213 (0.0003)	0.3894 (0.0432)	0.1555 (0.0020)
22	11.17	0.2775 (0.0546)	0.1221 (0.0003)	0.3931 (0.0420)	0.1556 (0.0020)
24	9.55	0.2694 (0.0612)	0.1177 (0.0003)	0.3839 (0.0509)	0.1521 (0.0020)
25	8.56	0.2745 (0.0645)	0.1197 (0.0003)	0.3894 (0.0515)	0.1529 (0.0020)
26	12.00	0.2725 (0.0528)	0.1202 (0.0003)	0.3927 (0.0397)	0.1532 (0.0020)

*Note*: Sample size is averaged across all loci and accounts for missing data.

Abbreviations: uH_e_, unbiased expected heterozygosity; *π*, nucleotide diversity; SE, standard error.

### 3.4 Genotype‐climate associations

We sought to determine whether climate could help identify patterns of genomic diversity and structure. The full RDA model [genotypes ~ Conditional (UTM northing + UTM easting) + AET + CWD + PPT + Tmn] explained 1.0% of the total observed genetic variation (adjusted *R* ^2^ = 0.00957, *p* < .001). There were four significant RDA axes (*p* < .001) and outlier analysis identified 1270 putatively adaptive SNPs that loaded ±3 SD from the mean loading on one or more axes. In total, these outlier SNPs exhibited higher genetic diversity (Table 3), greater differentiation across sites (global *F*_ST_ = 0.099, 95% CIs = 0.096 to 0.102), and significant isolation by distance (mantel test *r* = 0.6731, *p* < .001). Expected heterozygosity and nucleotide diversity of the outlier SNPs were positively correlated with latitude (uH_e_: *r* = .513, *p* < .015; *π*: *r* = .822, *p* < .001). Based on the strongest correlation between each outlier locus and climate variable, 494 of the identified outlier SNPs were most strongly associated with CWD, 283 with Tmn, 261 with AET, and 232 with PPT.

**FIGURE 7:**
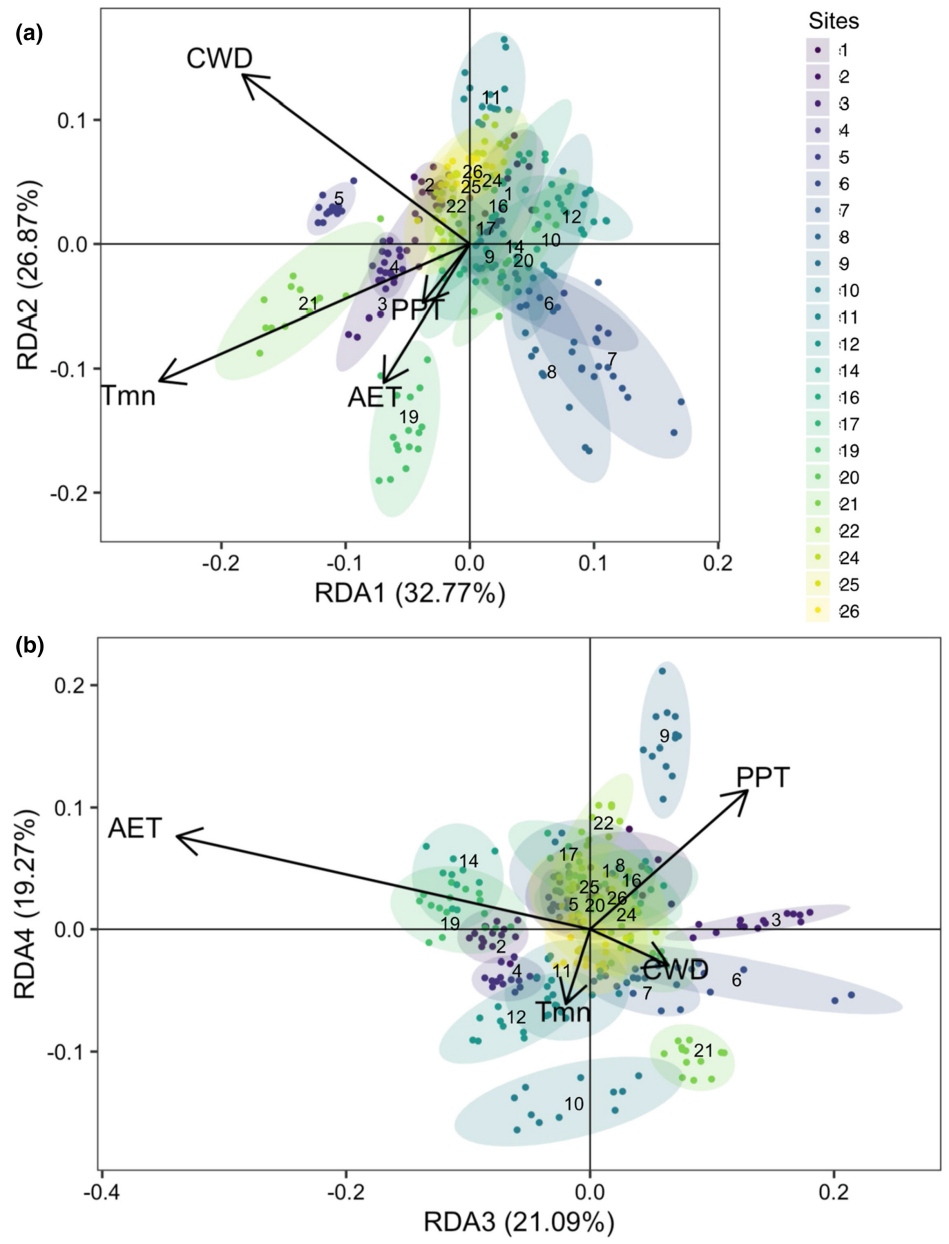
Redundancy analysis (a) axes 1 and 2, and (b) 3 and 4, where each point represents an individual multilocus genotype, color indicates site identity, and color gradient corresponds to site latitude (north to south; dark to light). Ellipses are 95% confidence intervals by site with label offset from ellipse center for legibility. Arrow direction and magnitude indicate how each significant climate variable loads onto the axes. Proximity of a genotype point to a climate vector indicates the strength of the association.

Individual trees showed variable loadings within and among sites with respect to the RDA axes, with some sites more tightly clustered and others displaying greater variation and overlap. This suggests some sites may contain a greater adaptive range associated with certain climate factors, but a much tighter range in others (Figure 7). Individual BAI drought response indices were correlated with RDA3 (*r*_*s*_ = .12, *p* = .05) and RDA4 (*r*_*s*_ = .13, *p* = .03). RDA3 appeared to differentiate high and low AET, with individuals in sites 3, 6, and 21 exhibiting the highest genotype loading and individuals from sites 14 and 19 lowest (Figure 7b). In addition, site 6 contained the highest variance in RDA3 scores. RDA4 differentiated lower PPT (sites 10, 21) from higher PPT (site 9; Figure 7b).

## 4 DISCUSSION

The objectives of our study were to describe how growth of whitebark pine in the Sierra Nevada responded to variability in climate (including meteorological drought) and to better understand patterns of genetic variation associated with this response. Whitebark pine at our sites generally had positive to neutral recent stem growth trends between 1970 and 2011. Stem growth, as measured by sBAI, appeared to be weakly positively correlated with water year annual minimum temperature and precipitation. Counterintuitively, growth indices during the meteorological drought years for BAI were generally positive to neutral. This implies that on average whitebark pine was not strongly moisture limited during the meteorological drought years (2012 to 2015). Individual growth response phenotypes appeared to be linked to genotypic variation in climate‐associated loci, indicating that drought growth response was not only controlled by site‐level environmental differences, but that some genotypes can take better advantage of local climatic conditions than others, as suggested in Millar et al. (2012).

We speculate that average stem growth, as measured by BAI drought response index, may have increased during the drought years due to an extended growing season. Whitebark pine in the Sierra Nevada often occupies energy‐limited environments, characterized by low minimum temperatures and often high snowpack. Warm air temperatures during the drought years may have been related to warm soil temperatures, potentially providing additional days where soil water was unfrozen and available to support photosynthesis. It may also be possible that snowpack losses during the drought years lengthened the growing season while still retaining sufficient moisture to permit growth. At Crater Lake National Park in Oregon, whitebark pine radial growth was negatively associated with instrumental records of snowpack (Jules et al., 2016). The mismatch between meteorological and agricultural drought (vegetation responses to moisture stress, such as reduced growth) is consistent with a number of other studies that specifically measured responses across environmental gradients. Lebourgeois et al. (2010) found that *Abies alba* and *Picea abies* were not strongly negatively affected by extreme drought in high‐elevation stands in France. Linares and Tíscar (2010) found that older trees growing at higher elevations maintained almost steady basal area increment during drought events, and Marqués et al. (2016) found that *Pinus sylvestris* was not strongly drought sensitive at higher elevation sites. These studies, as well as ours, suggest that high‐elevation sites that are more energy limited than water limited may not experience water limitation during periods of low precipitation (i.e., meteorological drought). Additional analyses using ring‐width data from whitebark pine at our sites suggest that most of our sampled sites were primarily energy limited (rather than water limited), supporting the idea that drought conditions could lead to increased stem growth (Dudney et al., In press). Future research that captures snow‐free days and soil moisture would provide insight into likely mechanisms driving growth patterns of whitebark pine in the Sierra Nevada.

### 4.1 Recent stem growth

We found increasing to neutral sBAI trends from 1970 to 2011. Similar trends have been reported elsewhere for trees near or at treeline in the Sierra Nevada (Graumlich, 1991; Millar et al., 2004, 2020; Salzer et al., 2009). Whitebark sBAI was positively correlated with annual water year temperature, with a smaller, positive effect of annual water year precipitation, in broad agreement with previous studies (Dolanc, Westfall, et al. 2013; Millar et al., 2012). While we found annual climatic predictors were statistically significant, these variables explained very little of the variation in site‐level average stem growth. There was high variation among individuals, perhaps reflecting intrinsic differences in tree genotypes (see below), tree size (see below), and microclimate (Bunn et al., 2011). Similar to our results, Millar et al. (2012, 2020) reported that annual growth of whitebark pine was positively correlated with precipitation at centennial scales (with varying relationships with temperature). Potentially important differences with these earlier studies and our results include length of measurement (e.g., Millar et al., 2020 used >100 years of tree‐ring records), site differences (eastern versus primarily western slope of the Sierra Nevada), and methodological differences (e.g., Millar et al., 2020 used detrended ring widths, while we used BAI without detrending). We emphasize the need for caution in extending our results to trees displaying *krummholz* growth forms, trees affected by pests and pathogens, and trees that did not survive the recent drought. That is, we cannot draw population‐level inferences from our samples because we did not exhaustively sample the population (Brienen et al., 2012). Future work may want to consider variability across tree conditions, growth forms, and live and dead trees to better describe population‐level growth responses to changing environmental conditions.

We found a negative relationship between sBAI and tree diameter. We believe this is not an artifact of sample design or data processing. Sample design, tree demography, and how growth is defined can strongly influence growth trends in tree‐ring data (Brienen et al., 2012, 2017; Nehrbass‐Ahles et al., 2014; Trouillier et al., 2020). At each site, non‐*krummholz* trees were sampled across a wide range of stem sizes and we focused on recent growth, so it is unlikely our results reflect a strong size or age bias. By normalizing each annual basal area increment by the standard deviation of the basal area increments within each tree, sBAI represents the relative direction and magnitude of the growth trend for each tree, independent of stem size. It is unclear whether the relationship between growth trends and stem size is intrinsic to tree size or age or reflects other variables covarying with size or age. Even the largest individual trees we sampled were short‐statured (maximum height = 22 m), so hydraulic limitations found in tall conifers are unlikely. Trees were often in open‐grown conditions, so changes in stand density were unlikely strongly biasing growth responses. One possibility is that larger older trees have established on sites with different conditions due to fine‐scale topographic mediation of snow depth and duration (Zald et al., 2012), and differential microsite conditions can result in different growth trends and growth–climate relationships (Oberhuber & Kofler, 2000). The importance and causes of size and age‐related growth trends in whitebark pine in the Sierra Nevada warrant further research but are beyond the scope of this study.

### 4.2 Stem growth during the drought years

Site‐average BAI was generally greater or unchanged during the drought years (2012 to 2015) relative to measurements prior to the drought years. We did not find evidence that the site‐averaged BAI response index was strongly variable across latitude, although the drought was more intense at lower latitudes in the Sierra Nevada (Goulden & Bales, 2019). The BAI drought response index was positively associated with latitude, but this pattern was largely driven by two sites at the northern portion of our sampling range. This lack of differentiation among sites for the drought response index may be partially explained by the generally low levels of genetic differentiation detected among sites (see below). The drought response index for BAI was negatively related to average water year CWD during the drought years. Correlations of drought response indices with climate should be viewed with caution; averaging annual variables over multiple years may obscure more subtle drivers of drought response, such as differences in plant phenology and seasonal timing of resource availability. Moreover, our sites likely included both energy‐ and water‐limited sites, where we expect different responses to drought (Dudney et al., In press).

### 4.3 Adaptive and neutral genomic structure

Despite low genetic differentiation overall, we detected significant associations between a subset of gene loci and climate. Our results indicate genetic associations related to differences in annual precipitation, minimum temperature, climate water deficit, and actual evapotranspiration. We observed higher heterozygosity and pairwise *F*_ST_ among study sites in this subset of loci indicating that this variation is more strongly structured across the sampling range. High levels of phenotypic variation in drought response among individuals both across and within plots appear to be correlated with at one or more axes of climate selection in whitebark pine. Notably, RDA3 and 4 were most strongly related to precipitation, AET and CWD, indicating genetic differences in outlier loci could occur along a moisture limitation gradient. There are several studies suggesting that water availability is a strong driver of genetic variation in whitebark and other pine species. For example, using similar tests of genetic environmental associations in whitebark pine from the Lake Tahoe Basin, Lind et al. (2017) found signatures of selection driven by water availability, with potential interactions between precipitation gradients and soil properties. Across the full species range, Syring et al. (2016) noted 12 functional genes with variants showing significant correlation with latitude. Although they found that heterozygosity was associated with geographic position rather than estimated climatic normals, the authors suggested these trends were likely driven by climate variation. Similarly, water availability was the main climatic driver of local adaptation in loblolly pine (*Pinus taeda*) surveyed across the southeastern United States (De La Torre et al., 2019). This study also concluded that adaptation to climate in loblolly pine has likely occurred due to changes in the frequency of alleles at many genes, each with small to moderate effects, as well as by alleles with large effects in fewer genes related to moisture deficit, temperature, and precipitation (De La Torre et al., 2019).

Genetic diversity was highest in northern sites and lowest in southern sites, a pattern that is consistent with a past range expansion (reviewed in Excoffier et al., 2009). A north‐to‐south diversity cline within the Sierra Nevada was also apparent in allozyme loci (Jorgensen & Hamrick, 1997). In the Sierra Nevada, high‐elevation whitebark pine range expansion may have occurred after the last major glacial retreat, ~12,000 to 13,000 years BP (Clark & Gillespie, 1997). Strong isolation by distance, low *F*_ST_, and weak structuring across a known sampling gap suggest that whitebark pine likely consists of a single genetic population across the Sierra Nevada. Relatively high genetic exchange across the Sierra Nevada likely facilitates a wide range of genetic variation and local adaptation to different environmental conditions (Tigano & Friesen, 2016). In particular, simulations suggest that when local adaptation is driven by many alleles of small effect size, good levels of standing variation in local populations are needed when gene flow is high (Yeaman, 2015). Maintaining gene flow may be an important consideration to help buffer against declines due to future climate change. Gene flow will likely be maintained through continued long‐distance seed dispersal by Clark's nutcracker and wind‐dispersed pollen, potentially assisting the dissemination of adaptive genotypes (Richardson et al., 2002; Rogers et al., 1999).

Gene–environment association studies represent an important step in understanding adaptation in natural populations of long‐lived species such as pines (De La Torre et al., 2019). We anticipate that our results could be used to develop sets of informative loci for widespread monitoring, with several additional steps. For example, mapping putative adaptive loci to annotated genomes would be useful in refining the dataset by determining the functional roles of gene variants. Until an annotated genome is published for whitebark pine, genomes of the closely related sugar pine, *Pinus lambertiana* (Crepeau et al., 2017), and loblolly pine (Neale et al., 2014) could provide a starting point (reviewed in Neale et al., 2017). Additionally, provenance tests (e.g., Chhin et al., 2018) or common garden studies (e.g., Warwell & Shaw, 2017) could be useful in determining the relative impacts of genetic and environmental factors underlying growth trends and variance, which are confounded in our approach. Provenance tests would also help to refine a set of screening loci associated with drought‐resilient genotypes for future restoration (sensu Axelsson et al., 2020). These studies could be particularly important in documenting seedling and early growth which may be vital stages in which mortality is high and selection is strong (Bower & Aitken, 2006). Our current dataset is limited in that it targeted only larger‐stemmed trees (≥6.3 cm DBH) and did not include *krummholz*. Expanded sampling and comparisons of seedling diversity to mature trees in stands could provide further information in diversity and adaptive trends over growth types and generations (Aravanopoulos, 2016).

### 4.4 Implications for conserving whitebark pine

The combination of population stability in some long‐term monitoring locations (Nesmith et al., 2019), neutral to positive growth trends in most sampling sites, and high genetic connectivity may be signs that whitebark pine was relatively healthy in specific regions of the Sierra Nevada. This contrasts with other parts of the species' range, where whitebark pine is in rapid decline (Goeking & Izlar, 2018; Keane et al., 2012). Increasing temperatures may be related to faster growth for Sierran whitebark pine, although future changes to growing conditions or more extreme drought may eventually reduce growth (Ullrich et al., 2018). Warming trends are also expanding the range of white pine blister rust into whitebark pine in the southern Sierra Nevada (Dudney et al., 2021). Long‐term projections of population stability will be unreliable without including likely shifts in pest and pathogen responses to forecasted warming.

Conserving Sierra Nevada whitebark pine is particularly important given that it represents a genetically distinct, southernmost population of a widely distributed species (Richardson et al., 2002). We expect whitebark pine in the Sierra Nevada to occupy sites that are generally warmer and drier relative to the majority of its range, potentially providing source material for recovery efforts under warming climates. Screening for rust resistance genotypes is ongoing in the Sierra Nevada and throughout the much of the range of whitebark pine (reviewed in King et al., 2010, Sniezko & Koch, 2017). Ultimately, disease and drought‐resistant genotypes could be preferentially planted in strategic areas to facilitate conservation and restoration (e.g., Landguth et al., 2017). Such strategies have been generally proposed for forest management in California (Young et al., 2020). However, much of the whitebark pine populations in the Sierra Nevada are in wilderness areas with more restricted opportunities for planting seedlings. Direct planting of seeds is an alternative action that may avoid some of these difficulties (Pansing & Tomback, 2019). Our results suggest that efforts to facilitate the spread of adaptive genotypes could include selection of individuals from a broad geographic region (e.g., Mahalovich & Hipkins, 2011). Detailed tracking of microsite conditions of seed sources and genomic screening once key genes for climate and other site conditions have been identified can help support restoration and conservation efforts of this imperiled species.

## AUTHOR CONTRIBUTIONS

**Phillip J. van Mantgem:** Conceptualization (equal); data curation (supporting); formal analysis (equal); funding acquisition (equal); investigation (equal); project administration (lead); writing – original draft (equal); writing – review and editing (equal). **Elizabeth R. Milano:** Conceptualization (equal); data curation (equal); formal analysis (equal); investigation (equal); visualization (equal); writing – original draft (equal); writing – review and editing (equal). **Joan Dudney:** Conceptualization (equal); data curation (equal); formal analysis (supporting); funding acquisition (equal); investigation (equal); methodology (equal); writing – original draft (supporting); writing – review and editing (equal). **Jonathan C. B. Nesmith:** Conceptualization (equal); data curation (equal); funding acquisition (equal); investigation (equal); methodology (equal); project administration (equal); resources (equal); supervision (equal); writing – review and editing (equal). **Amy G. Vandergast:** Conceptualization (equal); data curation (equal); formal analysis (supporting); funding acquisition (equal); investigation (equal); methodology (equal); project administration (equal); resources (equal); supervision (equal); writing – original draft (supporting); writing – review and editing (equal). **Harold S. J. Zald:** Data curation (equal); formal analysis (equal); investigation (equal); methodology (equal); resources (equal); supervision (equal); writing – original draft (supporting); writing – review and editing (equal).

## CONFLICT OF INTEREST STATEMENT

The authors declare no competing interests with this work.

## Supporting information


**TABLE S1** Comparisons of stringent filtering parameters on dataset characteristics and estimates of population genetic parameters. All filters had minor effects on the number of loci, number of samples, and no effect on estimates of population structure in the Sierra Nevada. Filtering out individuals with <80% data completeness reduced the number of individuals by almost half and was not explored further.

## Data Availability

The data for this study are available on ScienceBase (https://www.sciencebase.gov/catalog/item/6154a72fd34e0df5fb9d871b).
