# Supplementary material for: Growth, drought response, and climate‐associated genomic structure in whitebark pine in the Sierra Nevada of California
Source: Ecol Evol. 2023 May 17;13(5):e10072. doi: 10.1002/ece3.10072 (PMC10191741; doi:10.1002/ece3.10072)
Supplement: Supplementary file 1 — TABLE S1 Comparisons of stringent filtering parameters on dataset characteristics and estimates of population genetic parameters. All filters had minor effects on the number of loci, number of samples, and no effect on estimates of population structure in the Sierra Nevada. Filtering out individuals with <80% data completeness reduced the number of individuals by almost half and was not explored further. [file ECE3-13-e10072-s001.docx]

Table S1: Comparisons of stringent filtering parameters on dataset characteristics and estimates of population genetic parameters. All filters had minor effects on the number of loci, number of samples, and no effect on estimates of population structure in the Sierra Nevada. Filtering out individuals with less than 80% data completeness reduced the number of individuals by almost half and was not explored further.

| **Metric** | **Full** | **MAF 0.05** | **Ind >70%** | **MAF 0.05 & Ind >70** | **Ind >80%** |
| --- | --- | --- | --- | --- | --- |
| No. of loci | 44287 | 44070 | 44287 | 44070 | 44287 |
| No. of samples | 327 | 327 | 262 | 262 | 178 |
| Min. minor allele freq. | 0.02 | 0.05 | 0.012 | 0.05 | 0.003 |
| Avg. missing data | 0.2 | 0.2 | 0.16 | 0.16 | 0.12 |
| Max. missing ind. | 0.45 | 0.45 | 0.30 | 0.30 | 0.2 |
| Max. missing loc. | 0.36 | 0.36 | 0.40 | 0.40 | 0.44 |
| Avg. pairwise F_ST_ Sierra | 0.049 | 0.049 | 0.049 | 0.049 | NA |
| K Sierra | 2,3 | 2,3 | 2,3 | 2,3 | NA |
| Avg uHe Sierra | 0.279 | 0.280 | 0.279 | 0.280 | NA |
| Avg F_IS_ Sierra | 0.063 | 0.063 | 0.063 | 0.063 | NA |
